# Kynurenine aminotransferase activity of Aro8/Aro9 engage tryptophan degradation by producing kynurenic acid in *Saccharomyces cerevisiae*

**DOI:** 10.1038/s41598-017-12392-6

**Published:** 2017-09-22

**Authors:** Kazuto Ohashi, Romanas Chaleckis, Masak Takaine, Craig E. Wheelock, Satoshi Yoshida

**Affiliations:** 1 Institute for Molecular and Cellular Regulation, Gunma University, Gunma, Japan; 2 Gunma University Initiative for Advanced Research (GIAR), Gunma University, Gunma, Japan; 3 Division of Physiological Chemistry 2, Department of Medical Biochemistry and Biophysics, Karolinska Institutet, Stockholm, Sweden

## Abstract

Kynurenic acid (KA) is a tryptophan (Trp) metabolite that is synthesised in a branch of kynurenine (KYN) pathway. KYN aminotransferase (KAT) catalyses deamination of KYN, yielding KA. Although KA synthesis is evolutionarily conserved from bacteria to humans, the cellular benefits of synthesising KA are unclear. In this study, we constructed a KAT-null yeast mutant defective in KA synthesis to clarify the cellular function of KA. Amino acid sequence analysis and LC/MS quantification of KA revealed that Aro8 and Aro9 are the major KATs. KA was significantly decreased in the *aro8Δ aro9Δ* double mutant. We found that *aro8Δ aro9Δ* cells did not exhibit obvious defects in growth or oxidative stress response when proper amounts of amino acids are supplied in the media. We further found that *aro8Δ aro9Δ* cells were sensitive to excess Trp. The Trp sensitivity was not rescued by addition of KA, suggesting that Trp sensitivity is not due to the loss of KA. In conclusion, we propose that KAT activity is required for detoxification of Trp by converting it to the less toxic KA.

## Introduction

Kynurenic acid (KA) is a tryptophan (Trp) metabolite first identified in dog urine^1^. KA is present in several tissues and physiological fluids in humans^2–8^. KA has been shown to interact with the AMPA receptor, NMDA receptor, GPR35 receptor, AHR nuclear receptor, and sulfotransferases^9–17^. Although KA is found in unicellular organisms, such as bacteria and yeasts, its cellular function is still unclear.

**Figure 1:**
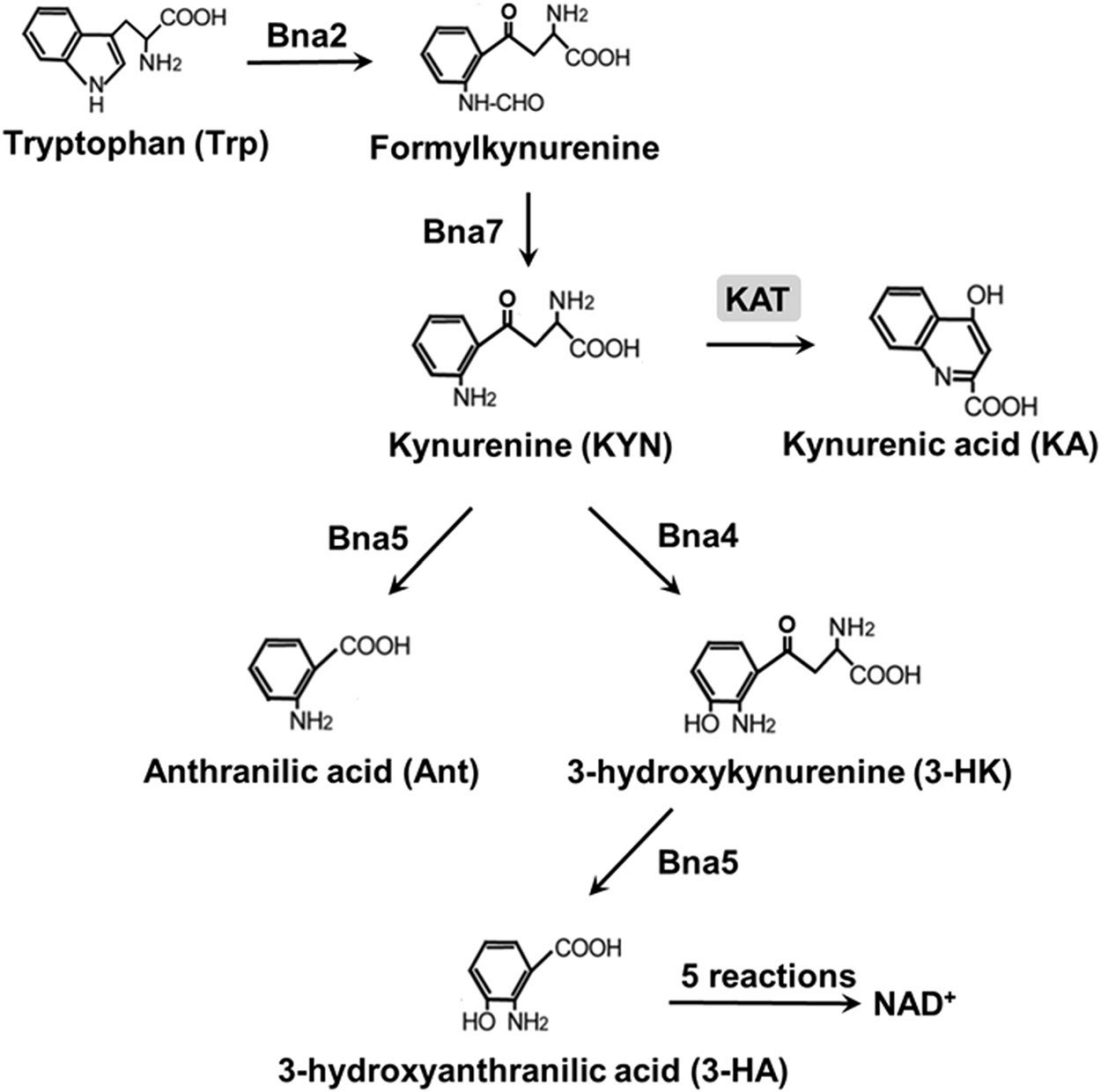
KYN pathway. KYN is synthesised from Trp by Bna2 and Bna7. Bna4, Bna5, and KAT convert KYN to Ant, 3-HK, and KA respectively.

KA is synthesised by kynurenine (KYN) aminotransferases (KATs), which catalyse irreversible deamination of KYN to KA in a branch of the KYN pathway. The KYN pathway is conserved from bacteria to humans for Trp catabolism and *de novo* synthesis of NAD^+^ (Fig. 1). NAD^+^ is a well-known coenzyme that is involved in many oxidation-reduction reactions. NAD^+^ is also a substrate in several reactions, including mono- and poly-ADP-ribosylation, cyclic ADP-ribose synthesis, and histone deacetylation^18–20^. NAD^+^ is synthesised not only via the KYN pathway but also via the salvage of NAD^+^ precursors, which are well-known vitamins such as nicotinic acid (NA) and nicotinamide. Also, nicotinamide ribose, nicotinamide mononucleotide, nicotinic acid ribose, and quinolinic acid have been identified as NAD^+^ precursors^21–27^. These NAD^+^ precursors are imported into the yeast cells from the media and are assimilated for the NAD^+^ supply^28,29^. On the other hand, Trp is also utilized for NAD^+^ supply via the KYN pathway. The first step in the pathway is catalysed by Bna2, which converts Trp to formylkynurenine^26^. The oxidation of formylkynurenine is catalysed by Bna7 to produce KYN^30^. KYN is then converted either to (i) 3-hydroxykynurenine (3-HK) by Bna4, (ii) anthranilic acid (Ant) by Bna5, or (iii) KA by KAT^26^. Thus, KYN is at a branch point of this pathway. 3-HK results in NAD^+^ through 3-hydroxyanthranilic acid (3-HA) (Fig. 1)^26,31^. However, the fate of KA is not known in *S. cerevisiae* or higher eukaryotes, although the degradation pathway was suggested in gram-negative bacterium *Pseudomonas fluorescens* ^32^.

In this study, we confirmed Aro8 and Aro9 as primary KATs in *S. cerevisiae* and showed that the *aro8Δ aro9Δ* mutant cells lack KA. Second, we showed that *aro8Δ aro9Δ* cells were sensitive to high concentrations of Trp. While expression of *ARO9* rescued the sensitivity for Trp in the *aro8Δ aro9Δ* double mutant, addition of KA to the medium did not. These data suggested that KAT activity served to degrade Trp to KA, which is relatively less toxic.

## Results

### KA was not used as a NAD^+^ precursor

NAD^+^ is synthesised via the KYN pathway (Fig. 1). *S. cerevisiae* cells lacking *BNA2* (*bna2Δ* cells) are inviable in the medium without NAD^+^ precursors, such as NA or nicotinamide, because they lack the ability to synthesise NAD^+^^23,26^. We tested whether KA and KYN serve as NAD^+^ precursors in the yeast. The growth of *bna2Δ* cells in the presence of KA or KYN was examined, and only KYN, but not KA, rescued the growth of *bna2Δ* cells (Supplementary Fig. S1). Thus, KA is unlikely to be used as a NAD^+^ precursor in the yeast.

### Identification of KATs in budding yeast

**Figure 2:**
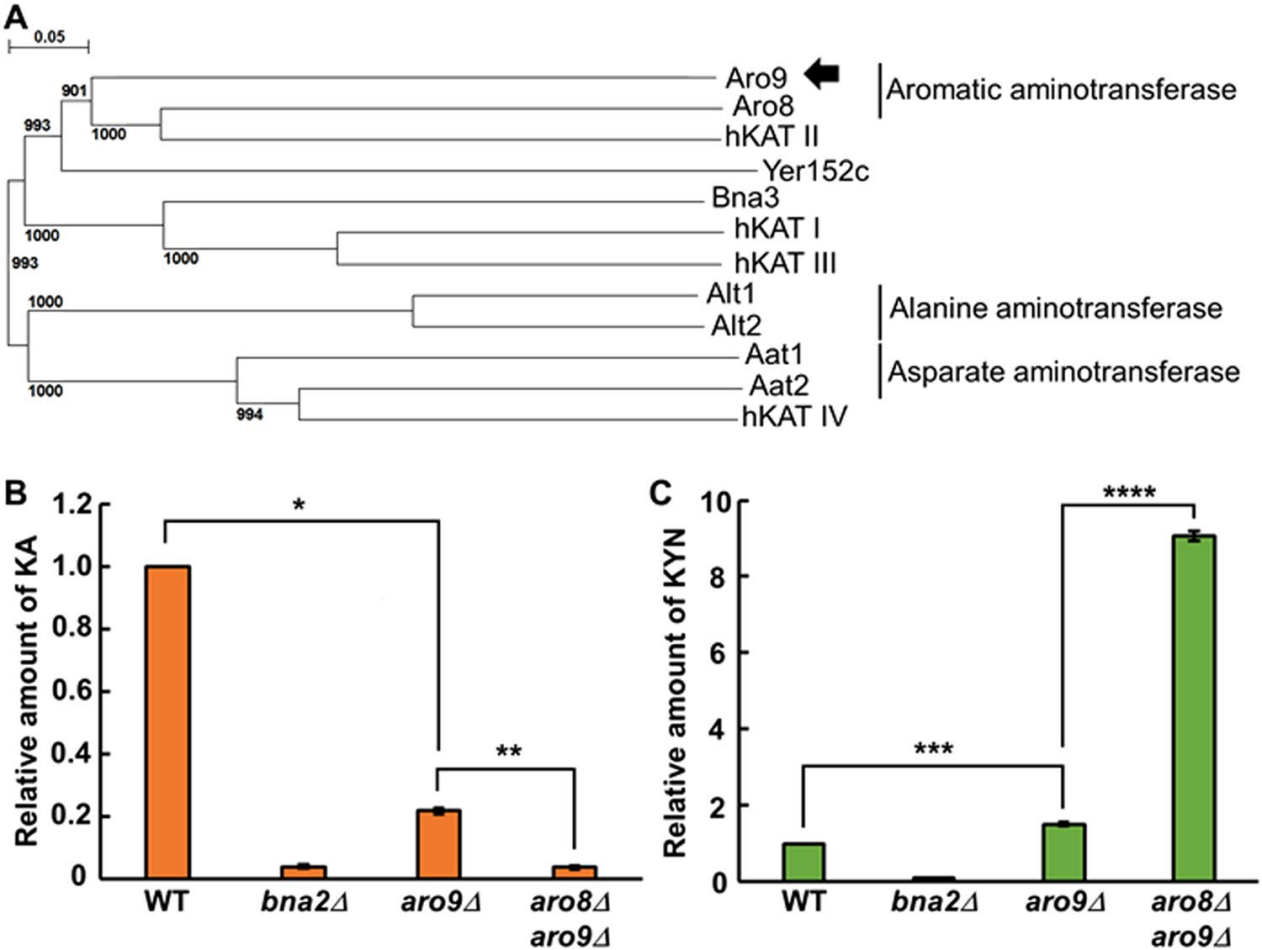
Identification of *S. cerevisiae* KATs for constructing a KA-lacking mutant. (**A**) Phylogenetic tree of *S. cerevisiae* KAT candidates and human KATs. Bootstrap values are indicated on branches. (**B**) LC/MS quantification of intracellular KA levels in the cell extract of the indicated mutants. The values of KA levels are WT: 1, *bna2Δ*: 0.04, *aro9Δ*: 0.22, and *aro8Δ aro9Δ*: 0.04. (**C**) LC/MS quantification of intracellular KYN levels in the cell extract of the indicated mutants. The values of KYN levels are WT: 1, *bna2Δ*: 0.1, *aro9Δ*: 1.5, and *aro8Δ aro9Δ*: 9.1. (**B** and **C**) Statistical analysis was performed by Welch’s *t*-test; *p = 0.0008, **p = 0.0004, ***p = 0.0009, ****p = 0.00002. Standard error of the mean is shown by error bars (n = 3).

**Figure 3:**
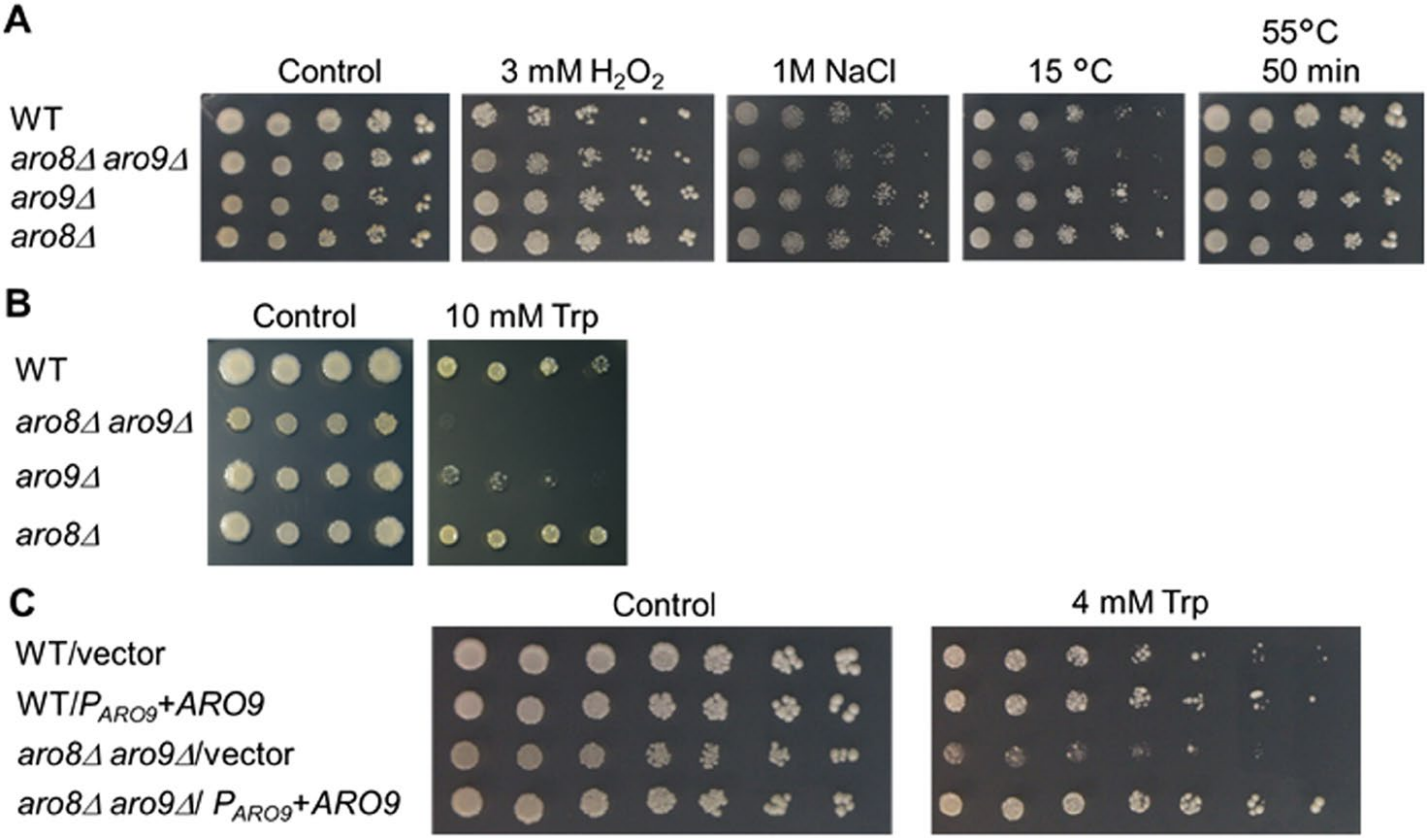
Growth phenotypes of *aro8Δ aro9Δ* cells. SC media with 30 °C incubation was used as an unstressed condition (Control). (**A**) Growth of *aro8Δ aro9Δ* cells on various stress conditions. The indicated cells were grown for 2–3 days under the stress conditions. (**B**) Trp sensitivity of *aro8Δ aro9Δ* cells. The indicated cells were grown for 4–5 days in the absence or presence of 10 mM Trp. (**C**) *ARO9* expression complemented the Trp sensitivity of *aro8Δ aro9Δ* cells. The indicated cells were grown for 4–5 days in the absence or presence of 4 mM Trp.

KATs catalyse the transfer of amine residues from KYN to an alpha keto acid and produce KA. KAT activity was reported in *in vitro* assays using cell lysates of *S. cerevisiae* ^33,34^. A previous report suggested that Bna3, Aro8, and Aro9 have KAT activity^30^. However, it is unclear whether they are the only KATs in the yeast. To identify the enzyme responsible for KA production in the yeast cells, we tried to re-identify the primary KAT *in silico* and *in vivo*. KATs have been extensively studied in mammals. Four KATs, KAT I (glutamine transaminase K/cysteine conjugate beta-lyase I), KAT II (aminoadipate aminotransferase), KAT III (cysteine conjugate beta-lyase 2), and KAT IV (glutamic-oxaloacetic transaminase 2/mitochondrial aspartate aminotransferase) have been identified and characterised in humans^35–38^. We performed a BLASTP^39,40^ search using the amino acid sequences of human KATs (KAT I, KAT II, KAT III, and KAT IV) as a query. When the BLASTP search was conducted with KAT I, KAT II, or KAT III as a query, 6 proteins (Bna3, Aro9, Aro8, Yer152c, Alt1, and Alt2) were detected as common hits (E-value cut-off, 0.01). When a BLASTP search was conducted with KAT IV as the query, 2 aminotransferases (Aat1 and Aat2) were identified (E-value cut-off, 0.01). Sequence analysis and phylogenetic tree analysis revealed that these 8 proteins contain a common motif (Pfam, pf: Aminotran_1_2) and are predicted as aminotransferases. However, it was difficult to determine the substrate specificity for KYN from their sequence analyses (Fig. 2A, Supplementary Fig. S2). To identify KATs in *S. cerevisiae*, therefore, we measured the concentration of KA and KYN in the cell extract from mutant cells with a gene deletion in KAT candidates (*bna3Δ*, *aro8Δ*, *aro9Δ*, *aat1Δ*, *alt1Δ*, *alt2Δ*, and *yer152cΔ*) by LC/MS. KA was reduced only in *aro9Δ* cell extract (Fig. 2B). Since low levels of KA were still detected in *aro9Δ* cells, we further introduced deletion of the KAT candidate genes into the *aro9Δ* mutant. In the phylogenetic tree of *S. cerevisiae* KAT candidates and human KATs, Aro8 and Aro9 were separated into the same branch with human KAT II, indicating that Aro8 showed the highest similarity to Aro9 (Fig. 2A). Aro9 is 29% identical (51% positively similar amino acids and 13% gaps) to Aro8 and 27% identical (49% positively similar amino acids and 18% gaps) to human KAT II. Therefore, we constructed an *aro8Δ aro9Δ* double mutant and measured the concentration of KA in the cell extract of the mutant. KA was significantly reduced in *aro8Δ aro9Δ* cells to the same level as in *bna2Δ* cells, and KYN was accumulated in *aro8Δ aro9Δ* cells than in *aro9Δ* cells (Fig. 2B,C), indicating that Aro9 and Aro8 are major KATs that produce KA from KYN. The reduction of KA and accumulation of KYN was also confirmed in *aro9Δ* cells (Fig. 2B,C). Notably, KA levels in the *aro8Δ aro9Δ* cells were almost the same as those in *aro8Δ aro9Δ yer152c Δ* and *aro8Δ aro9Δ yer152cΔ bna3Δ* cells, and further reduction of KA was not detected in preliminary data (Supplementary Fig. S3). We also noticed that the levels of KYN are significantly higher in *aro8Δ aro9Δ yer152cΔ* and *aro8Δ aro9Δ yer152cΔ bna3Δ* cells compare with *aro8Δ aro9Δ* cells (Supplementary Fig. S3). The reason why KYN accumulate in *aro8Δ aro9Δ yer152cΔ* and *aro8Δ aro9Δ yer152cΔ bna3Δ* cells remains to be elucidated. We decided to use *aro8Δ aro9Δ* cells as the KA-lacking mutant, although we cannot eliminate the possibility of low amounts of remaining KA. We found that the growth of *aro8Δ aro9Δ* cells was normal on SC media with 30 °C incubation (unstressed condition) (Fig. 3A). This suggested that KA is not essential for cell growth.

### *aro8Δ aro9Δ* cells were sensitive to high doses of Trp

To elucidate the cellular function of KA, we investigated *aro8Δ aro9Δ* specific phenotypes. KA was reported to capture radicals and reduce reactive oxygen species^41–44^. We investigated whether *aro8Δ aro9Δ* cells were sensitive to hydrogen peroxide (H_2_O_2_). *aro8Δ aro9Δ* cells did not show obvious sensitivity to 3 mM H_2_O_2_ (Fig. 3A). We also tested several other stresses, including 1 M NaCl, heat shock at 55 °C for 50 min, and cold stress at 15 °C, but these conditions caused minor growth defects in *aro8Δ aro9Δ* cells (Fig. 3A, Supplementary Fig. S1).

Next, we focused on the role of KAT in the Trp degradation. We investigated whether excess Trp causes a problem in the growth of *aro8Δ aro9Δ* cells. As expected, *aro8Δ aro9Δ* and *aro9Δ* cells showed significant growth defects with 10 mM Trp compared to wild type (WT) cells (Fig. 3B). We confirmed that exogenous expression of Aro9 rescued the growth of *aro8Δ aro9Δ* cells on 4–5 mM Trp (Fig. 3C, Supplementary Fig. S4B). However, the growth deficiency of *aro8Δ aro9Δ* cells by 10 mM Trp was not rescued by adding 10 mM KA (Supplementary Fig. S4A). These data suggested that Aro8 and Aro9 activities, but not KA per se, are responsible for the tolerance to Trp. We observed that the growth of *aro9Δ* and *aro8Δ aro9Δ* cells was much slower than that of *aro8Δ* cells with 10 mM Trp, consistent with our hypothesis that Aro9 is the dominant KAT (Figs 2B and 3B).

### Detoxification of Trp and Ant by Aro8 and Aro9

**Figure 4:**
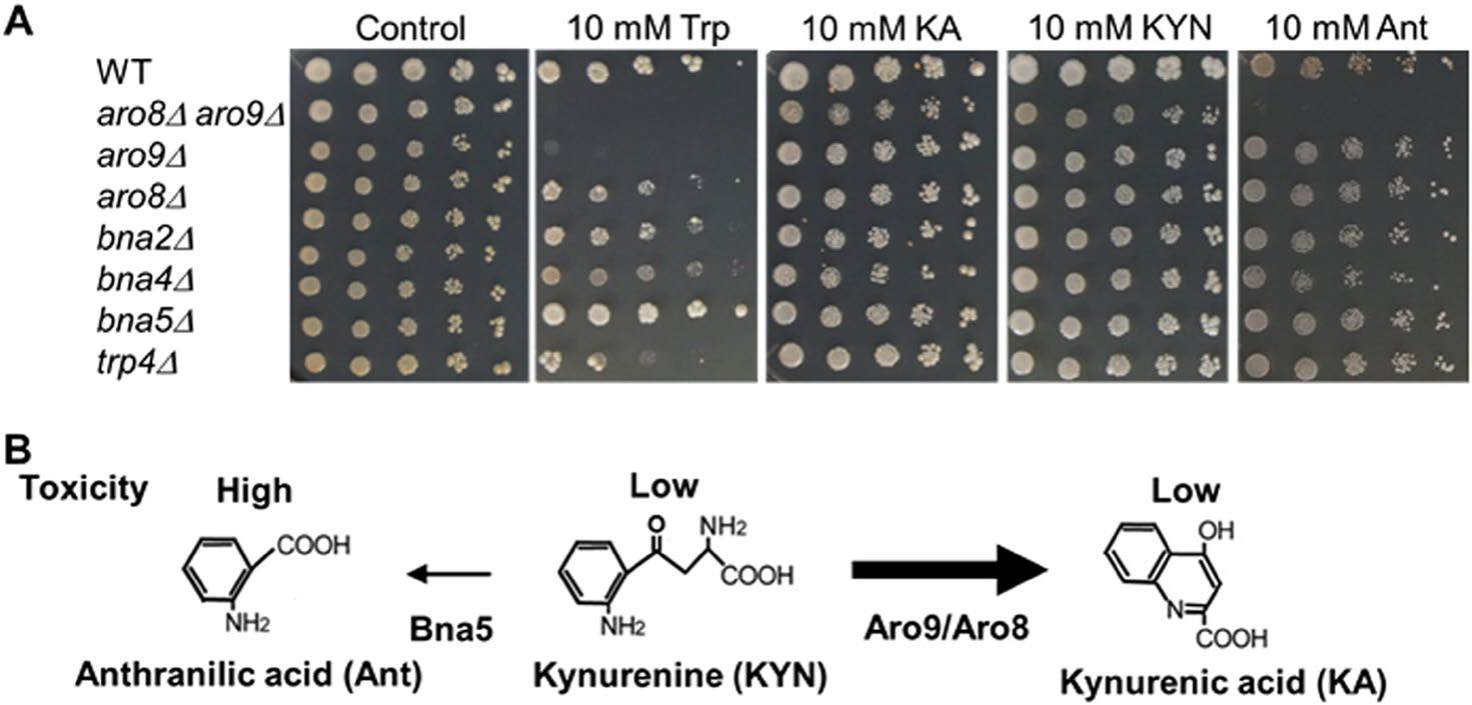
Growth phenotypes of KYN pathway-deficient mutants with high concentration of Trp and its metabolites. (**A**) Cells with the indicated genotypes were grown for 4–5 days in the absence or presence of 10 mM Trp, 10 mM KA, 10 mM KYN, or 10 mM Ant. (**B**) Schematic model for detoxification of Trp and Ant by KYN degradation to KA.

We hypothesised that a metabolite produced downstream of KYN pathway caused the toxic effects in *aro8Δ aro9Δ*. To identify the compound, we examined the growth phenotypes of the KYN pathway mutants on 10 mM Trp medium. *bna2Δ* cells did not show any sensitivity to 10 mM Trp medium (Fig. 4A). However, *bna4Δ* cells were slightly sensitive and *bna5Δ* cells were resistant to 6–10 mM Trp (Fig. 4A, Supplementary Fig. S4C,D). Additionally, we confirmed the accumulation of KYN in *bna4Δ* (Supplementary Fig. S3), which was suggested in a previous report^45^. Because Bna5 is responsible for Ant production from KYN, and Bna4 may reduce toxic Trp metabolites by consumption of KYN, we next examined the toxicity of Ant and KYN. We found that *aro8Δ aro9Δ* was sensitive to 10 mM Ant medium (Fig. 4A). In contrast, none of the mutants were sensitive to KYN or KA (Fig. 4A). These results suggest that toxic metabolites, including Ant, contribute to the toxicity of Trp (Fig. 4B). However, Ant is not the only reason why high dosage of Trp is toxic since Trp toxicity was not attenuated in *bna2Δ* cells defective in Ant synthesis.

## Discussion

KA is synthesised by KAT in a branch of the KYN pathway. In humans, four KATs have been identified and characterized^35–38^. Although KAT activity was biochemically detected in the yeast, it is unclear which gene is responsible for synthesising KA *in vivo* ^26,30,33,34^. In this study, endogenous KA was extracted from cells and quantified by LC/MS. Aro8 and Aro9 were identified as the major enzymes responsible for KA production in the yeast. We showed that *aro8Δ aro9Δ* cells were sensitive to Trp and Ant but not to KA and KYN. In mouse, KYN is believed to be neurotoxic, and KAT in the skeletal muscle protects against neural damage by converting KYN to KA^46^. Similarly, we propose that Aro9 and Aro8 reduce toxic Trp and its metabolites indirectly by converting KYN to KA in the yeast.

Although we focused on KA in this study, Aro8 and Aro9 also catalyse the deamination of Trp to produce indolepyruvate (Supplementary Fig. S5). Notably, Aro9 was reported to prefer Trp to tyrosine or phenylalanine as a substrate^33,34^. In the detoxification of Trp, Aro9 was more effective than Aro8 (Fig. 3B). Trp aminotransferase activity of Aro9 also should participate in Trp detoxification^47^. Consistent with our proposal that Aro9 is important for the detoxification of Trp, transcription of Aro9 was reported to be induced by Trp^34,48–50^.

Additionally, we found that *aro8Δ aro9Δ* was sensitive to high concentrations of Ant, although other mutants did not show sensitivity (Fig. 4A). Furthermore, *trp4Δ* cells, which accumulate Ant, were sensitive to Trp, and *bna5Δ* cells, which are defective in Ant synthesis, were resistant to Trp (Fig. 4A). These results identified Ant as one of the potential toxic metabolites produced by excess Trp. However, *trp4Δ* was found to be resistant to Ant (Fig. 4A), suggesting Ant is not the only metabolite that causes toxicity.

In conclusion, we propose that KAT activity primarily contributes to the degradation of excess Trp by converting it to the less toxic KA in the yeast. Given that KA is abundant in urine (in humans, the ratio of KA to KYN is 1.24 in urine^51^ and 0.02 in serum^52^), the role of KAT in detoxification of Trp may be a widely conserved mechanism.

## Methods

### Yeast cultivations

Standard media, such as yeast extract/peptone/dextrose (YPD) media, were used for the cultivation of *S. cerevisiae*. The optimised minimal medium, which was reported in Hanscho *et al*.^53^, supplemented with phenylalanine, tyrosine, and Trp was used as SC medium in this study. The detailed composition of 1 L of SC media included 6.7 g yeast nitrogen base (YNB) without amino acid and with ammonium sulfate (Thermo Fisher Scientific, Waltham, USA), 20 g glucose, 0.035 g L-histidine, 0.11 g L-leucine, 0.1 g L-glutamate, 0.12 g L-lysine, 0.04 g L-methionine, 0.1 g L-phenylalanine, 0.38 g L-serine, 0.2 g L-threonine, 0.03 g L-tyrosine, 0.02 g L-Trp, 0.04 g uracil, and 0.006 g inositol. For SC media without Trp and NA, Trp was removed from the composition, and 1.71 g/L YNB without NA (Sunrise Science Products, San Diego, USA) and 5 g/L ammonium sulfate was used instead of YNB. For solid media, 2% agar was added. Additional compounds were sterilised by a 0.2 μm filter and added to the media after autoclaving. Cells were grown aerobically at 30 °C in liquid or solid media. For analysis of the growth phenotype on solid media, 3.0 µl of serially diluted cell suspensions [3-fold serial dilution of cell suspension (*A*_600_ of 0.2) for Fig. 3B and C and Supplementary Fig. S4B and C, and 5-fold serial dilution of cell suspension (*A*_600_ of 0.2) for Figs 3A and 4A and Supplementary Figs S1, S4A, and S4D] were spotted onto the SC media containing the indicated compounds and grown at 30 °C under aerobic conditions. An appropriate nutrient was removed from SC media to maintain the plasmid.

### Plasmids and strains

A 2684 bp DNA fragment corresponding to the *ARO9* ORF flanked by the 625 bp upstream and the 517 bp downstream was amplified by PCR and cloned into SacI/XhoI sites of pSPG1, yielding *P*_*ARO9*_ + *ARO9*-pSPG1. Accurate synthesis of all the constructed plasmids was confirmed by DNA sequencing.

Yeast deletion strains (*aro8Δ*, *aro8Δ aro9Δ*, *aro8Δ aro9Δ yer152cΔ*, *aro8Δ aro9Δ yer152cΔ bna3Δ*, and *bna2Δ bna4Δ*) were constructed by PCR-based method using the *his3MX6*, *hphNT1*, or *natNT2* cassette^54^. Correct replacement was confirmed by PCR. Yeast strains used in this study were listed in Table 1.

**Table 1:** Yeast strains used in this study.

Strains	Descriptions	Resource
WT (BY4741^a^)	*MAT*a *his3-Δ200 leu2-Δ0 ura3-Δ0 met15-Δ0*	EUROSCARF
*bna2Δ*	BY4741 *bna2::kanMX4*	EUROSCARF
*bna4Δ*	BY4741 *bna4::kanMX4*	EUROSCARF
*bna5Δ*	BY4741 *bna5::kanMX4*	EUROSCARF
*trp4Δ*	BY4741 *trp4::kanMX4*	EUROSCARF
*aro8Δ*	BY4741 *aro8::natNT2*	This study
*aro8Δ aro9Δ*	BY4741 *aro8::kanMX4 aro9::hphNT1*	This study
*aro8Δ aro9Δ yer152cΔ*	*MAT*α *his3-Δ200 leu2-Δ0 ura3-Δ0 aro8::natNT2 aro9::hphNT1 yer152c::his3MX6*	This study
*aro8Δ aro9Δ yer152cΔ bna3Δ*	BY4741 *bna3::kanMX4 aro8::natNT2 aro9::hphNT1 yer152c::his3MX6*	This study
*bna2Δ bna4Δ*	BY4741 *bna2::kanMX4 bna4::hphNT1*	This study
WT/pSPG1	BY4741 carrying pSPG1	This study
*aro8Δ aro9Δ*/pSPG1	*aro8Δ aro9Δ* carrying pSPG1	This study
WT/*P*_*ARO9*_ + *ARO9*	BY4741 carrying *P*_*ARO9*_ + *ARO9*-pSPG^b^	This study
*aro8Δ aro9Δ*/*P*_*ARO9*_ + *ARO9*	*aro8Δ aro9Δ* carrying *P*_*ARO9*_ + *ARO9*-pSPG1^b^	This study

^a^BY4741 was used as WT in this study.

^b^2684 bp of DNA from 625 bp upstream to 517 bp downstream of *ARO9* ORF was cloned into multi-copy plasmid pSPG1.

### Extraction of KYN and KA

BY4741 WT, *bna2Δ*, *bna4Δ*, *aro9Δ*, *aro8Δ aro9Δ*, *aro8Δ aro9Δ yer152cΔ*, and *aro8Δ aro9Δ yer152cΔ bna3Δ* cells were diluted to *A*_600_ of 0.1 in SC media and grown aerobically at 30 °C for 9 h for Fig. 2B and C, and Supplementary Figure S3. Cells were collected following centrifugation (2,000 × *g*, for 5 min) and quenched by adding pre-cooled (−20 °C) methanol with internal standards. The extraction method was modified for the yeast cells and performed as described^55^.

### LC/MS quantification of KYN and KA

Measurement of metabolites by LC/MS was performed using the all ion fragmentation (AIF) method as described in Naz *et al*.^56^. Briefly, yeast cell extracts were measured using an Agilent Ultra-high-performance liquid chromatography (UHPLC) 1290 Infinity II system coupled to a 6550 iFunnel quadrupole-time time of flight (Q-TOF) mass spectrometer (Agilent Technologies, Santa Clara, USA). Metabolites were separated using a HILIC SeQuant® ZIC®-HILIC column (100 mM × 2.1 mM, 100 Å, 3.5 µm, Merck, Darmstadt, Germany), with a gradient between water [containing 0.1% formic acid (v/v)], and acetonitrille [containing 0.1% formic acid (v/v)]. Database-dependent metabolite screening was performed; the identities of KA and KYN were confirmed by accurate mass, retention time, MS/MS fragments and ion ratios relative to authentic standards. For relative quantification of the KA and KYN, the peak areas of the precursor ions ([M + H]^+^) were used. Another method described below was used for Supplementary Fig. S3. The LCMS-8050 system (Shimazu) equipped with a Discovery HS F5-3 Column (2.1 mM × 150 mM, Sigma-Aldrich) was used. The mobile phase consisted of A [0.1% formic acid (v/v) in water] and B [0.1% formic acid (v/v) in acetonitrile]. Separation was achieved using the appropriate gradient from 100% A (v/v) to 5% A (v/v) and 95% B (v/v). The column was re-equilibrated with 100% A (v/v) for 5 min. The flow rate was 0.25 ml/min. Electrospray ionization was performed in a positive ion mode. Identification of KA and KYN was carried out using standards (KA and KYN from Sigma) with accurate mass, retention time, and MS/MS fragments.

### Analysis of the primary structure

For identification of an Aro9 homologue, we used the BLASTP^39,40^ program at KEGG (Kyoto Encyclopedia of Genes and Genomes website, http://www.genome.jp/kegg/). Multiple alignment of human KAT II, Aro8, and Aro9 was carried out by ClustalX 2.1^57^. The phylogenetic tree was constructed using the tool for rooted phylogenetic trees with branch length (also by ClustalX 2.1) and drawn by NJprot^58^.

### Data availability

The data supporting the findings of this study are included in this article and Supplementary Information.

## Electronic supplementary material

Supplementary Information
