# Supplementary material for: Kynurenine aminotransferase activity of Aro8/Aro9 engage tryptophan degradation by producing kynurenic acid in Saccharomyces cerevisiae
Source: Sci Rep. 2017 Sep 22;7:12180. doi: 10.1038/s41598-017-12392-6 (PMC5610271; doi:10.1038/s41598-017-12392-6)
Supplement: Supplementary file 1 — Supplementary Information [file 41598_2017_12392_MOESM1_ESM.pdf]

Supplementary Information

Article title: Kynurenine aminotransferase activity of Aro8/Aro9 engage tryptophan degradation by producing kynurenic acid in *Saccharomyces cerevisiae*

Authors: Kazuto Ohashi[1, *], Romanas Chaleckis[2, 3], Masak Takaine[1, 2], Craig E. Wheelock[2, 3], and Satoshi Yoshida[1, 2]

Affiliations: [1]Institute for Molecular and Cellular Regulation, Gunma University, Gunma, Japan

[2]Gunma University Initiative for Advanced Research (GIAR), Gunma University, Gunma, Japan

[3]Division of Physiological Chemistry 2, Department of Medical Biochemistry and Biophysics, Karolinska Institutet, Stockholm, Sweden

*Address correspondence to

Kazuto Ohashi, Ph.D.

Gunma University Institute for Molecular and Cellular Regulation

3-39-15 Showa-machi, Maebashi, Gunma 371-8512, Japan

Phone Number: +81-27-220-8881

Email: ohashi@gunma-u.ac.jp.

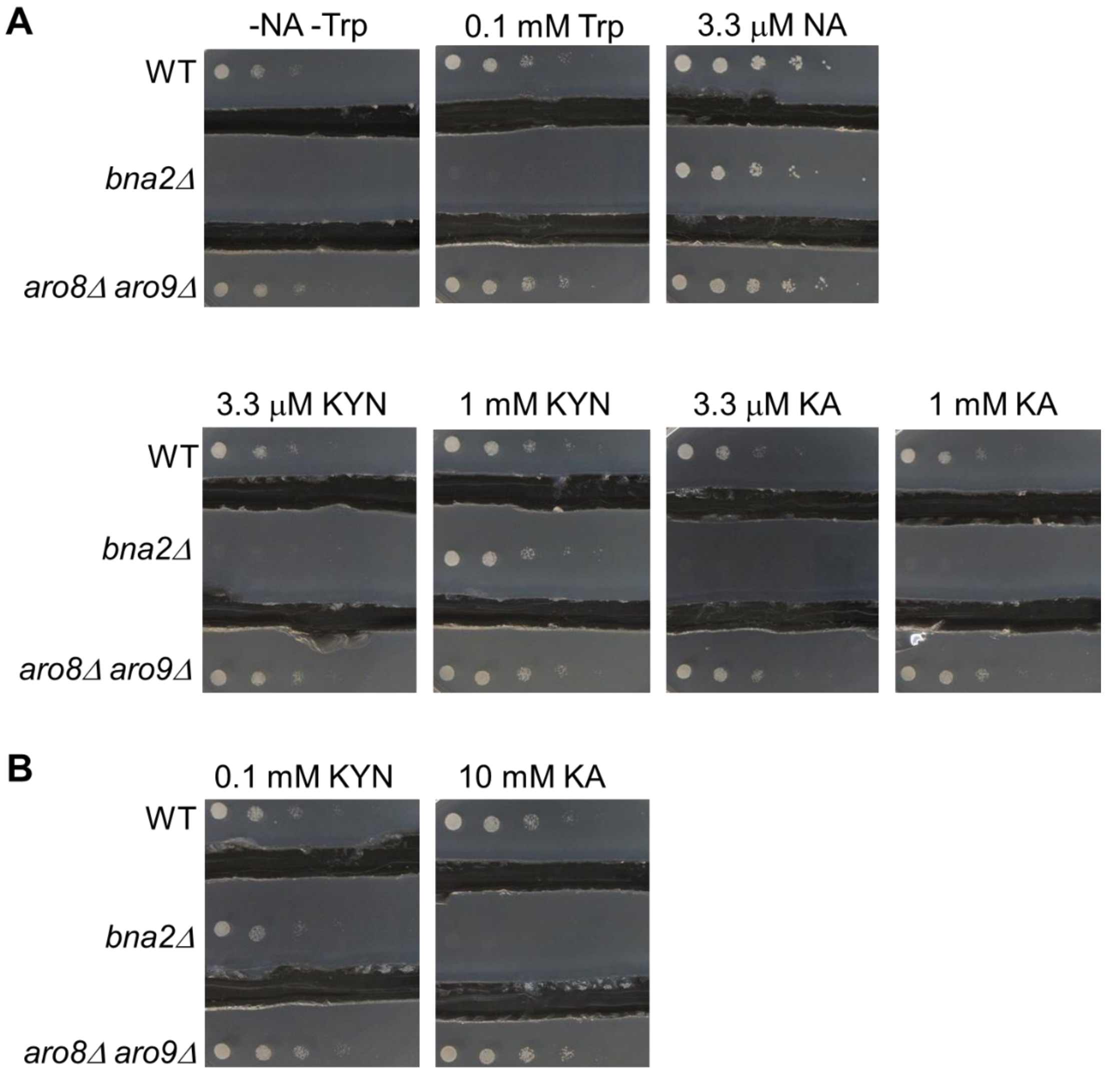

Supplementary Figure S1 | Growth phenotypes of yeast cells in the presence or absence of indicated compounds. Indicated compounds are added to SC media without Trp and Nicotinic acid (NA). NA was used as a control. The detailed composition of SC medium without Trp and NA was described in MATERIALS. The cleavages between strains were made for excluding the possibility of the NAD^+ precursor secretions[1,2]. Cells were grown at 30°C for 2-3 days.

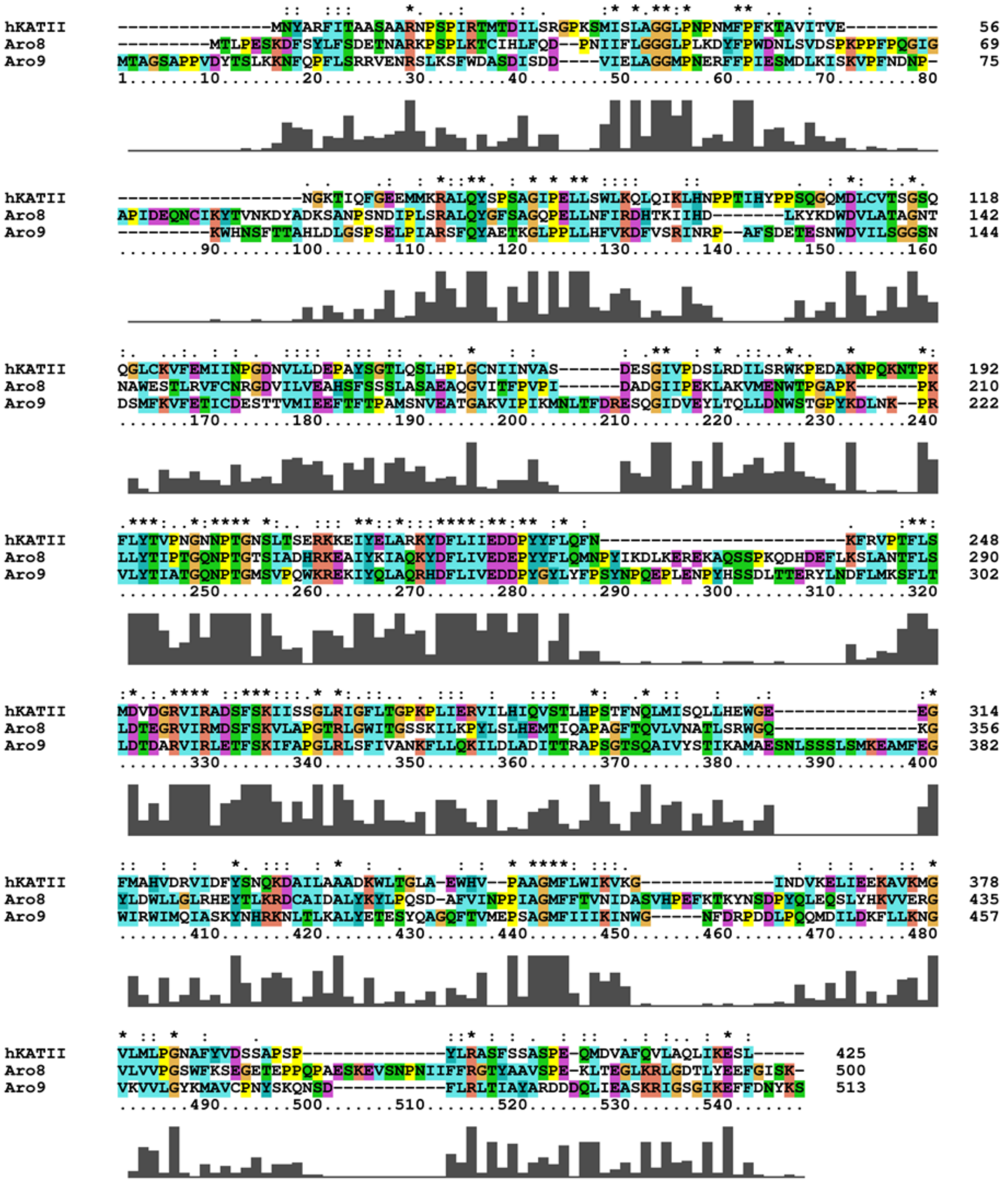

Supplementary Figure S2 | Multiple alignment of humanKATII, Aro8, and Aro9. Aligment was carried out by ClustalX 2.1.

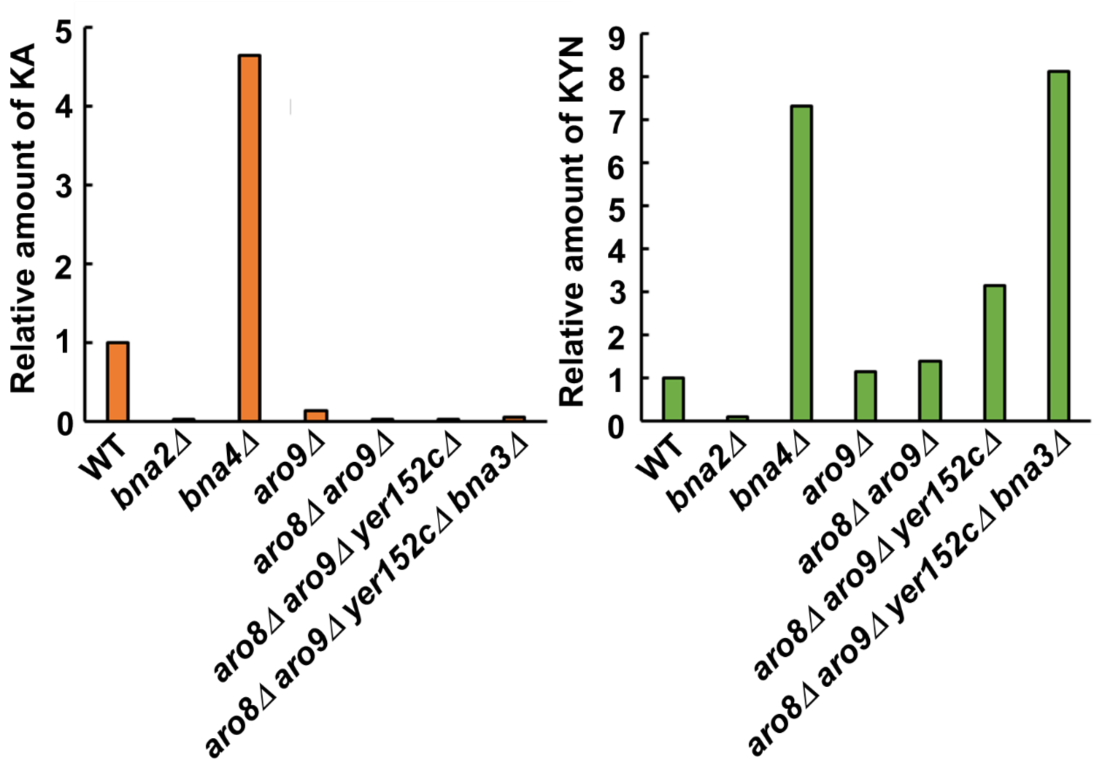

Supplementary Figure S3 | LC/MS measurements of KA and KYN in wild type, *bna2Δ*, *bna4Δ*, *aro9Δ*, *aro8Δ aro9Δ*, *aro8Δ aro9Δ yer152cΔ*, and *aro8Δ aro9Δ yer152cΔ bna3Δ* cells. Cells were grown at 30°C in SC medium. The detailed composition of SC medium was described in MATERIALS.

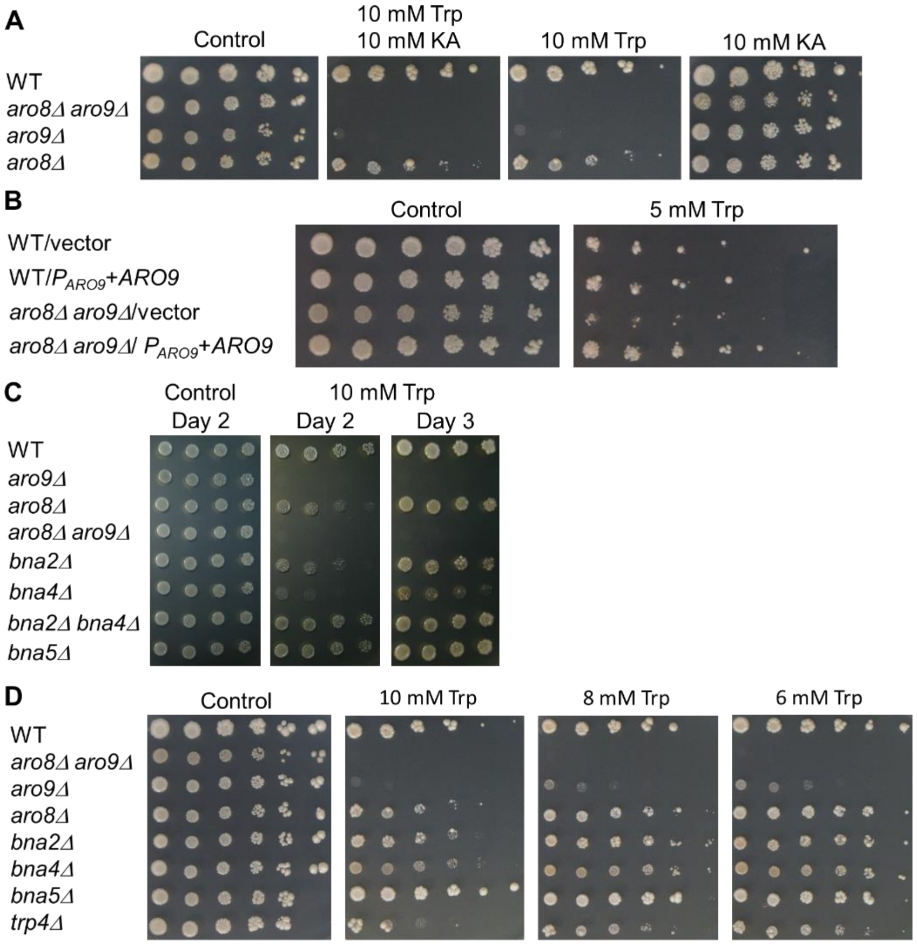

Supplementary Figure S4 | Aro9 activity was suggested to participate in detoxification of Trp. SC media was used for control. Detailed composition was described in MATERIALS. A. The effect of supplemental KA to the Trp sensitivity of *aro8Δ aro9Δ* cells. Indicated cells were grown at 30°C for 4-5 days. B. *ARO9* expression complement the Trp sensitivity of *aro8Δ aro9Δ* cells. Indicated cells were grown for 4-5 days in the absence or presence of 5 mM Trp. C. Trp sensitivity of kynurenine pathway deficient mutants. Indicated cells were grown at 30°C for 2 or 3 days. The growth of *bna4Δ* cells were slightly slower than wild type on Day 3. The Slow growth on 10 mM Trp was partially rescued by *BNA5* deletion on Day2. D. Trp sensitivity of kynurenine pathway deficient mutants. Indicated cells were grown at 30°C for 4-5 days.

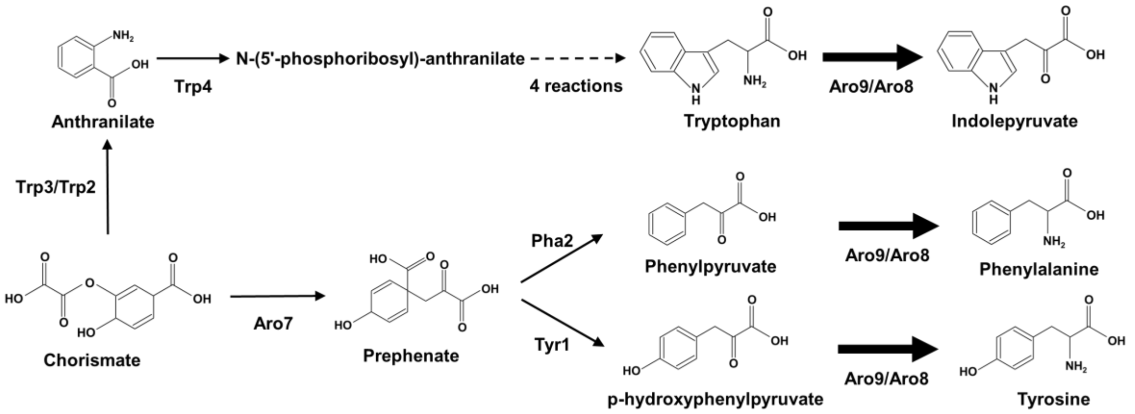

Supplementary Figure S5 | Aro8 and Aro9 are participate in biosynthetic pathway of Trp, Phe, and Tyr.

REFERENCES IN SUPPLEMENTAL INFORMATION

1 Lu, S. P. & Lin, S. J. Phosphate-responsive signaling pathway is a novel component of NAD+ metabolism in Saccharomyces cerevisiae. *J Biol Chem* **286**, 14271-14281 (2011).

2 Ohashi, K., Kawai, S. & Murata, K. Secretion of quinolinic acid, an intermediate in the kynurenine pathway, for utilization in NAD+ biosynthesis in the yeast Saccharomyces cerevisiae. *Eukaryot Cell* **12**, 648-653 (2013).
